# Supplementary material for: Saccharomyces boulardii (CNCM I-745) improves intestinal damage in sepsis by remodeling intestinal flora structure
Source: Front Cell Infect Microbiol. 2025 Jul 31;15:1602792. doi: 10.3389/fcimb.2025.1602792 (PMC12350329; doi:10.3389/fcimb.2025.1602792)
Supplement: Supplementary file 1 [file DataSheet1.zip › summary/summary/8_Advanced_analyse/2_PICRUSt2_result/KEGG/Group.CW4_vs_CW0/PICRUSt2_KEGG_level3_Group.CW4_vs_CW0_diff.pdf]

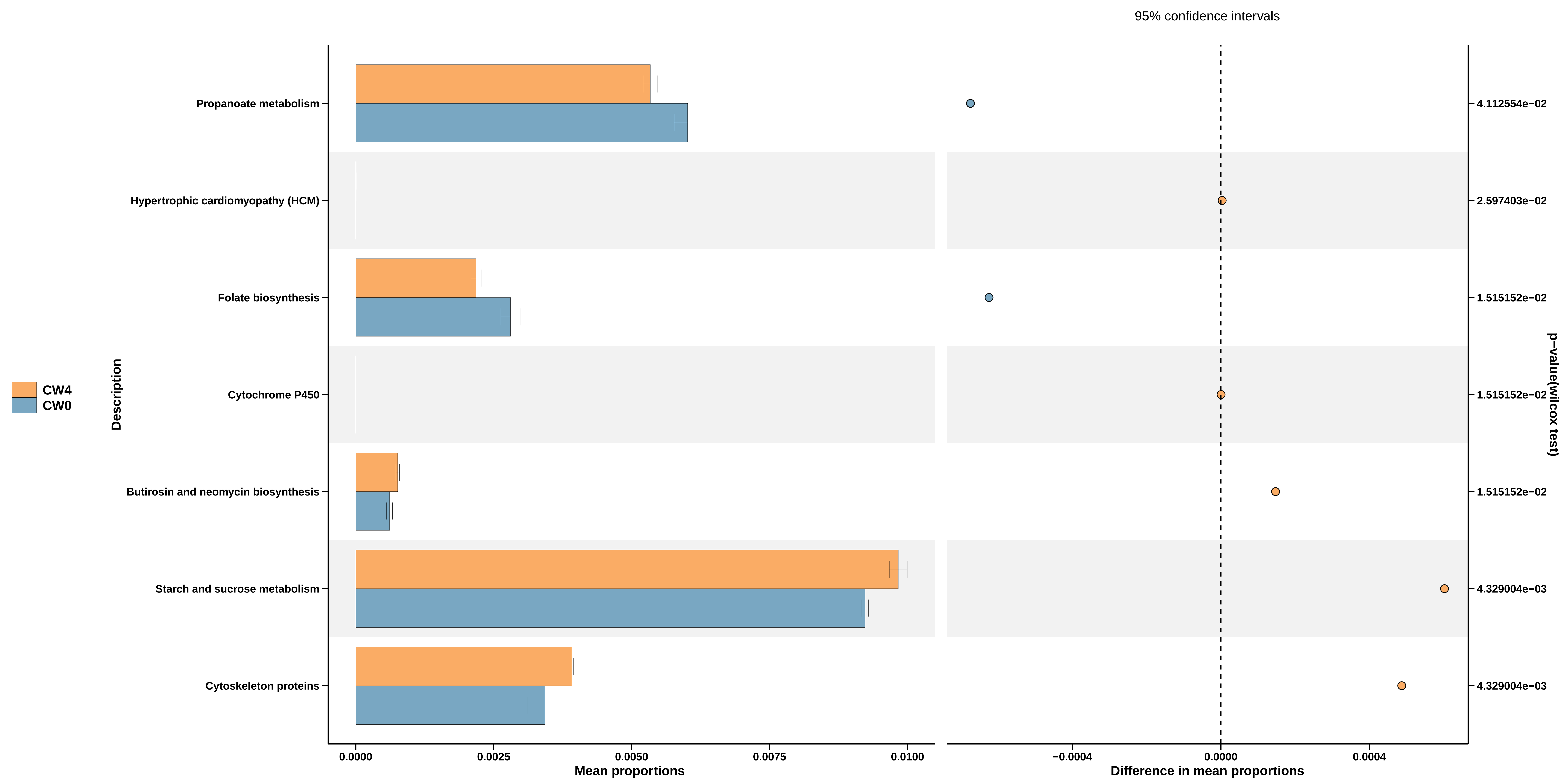

95% confidence intervals
CW4
CW0
Description
Propanoate metabolism
Hypertrophic cardiomyopathy (HCM)
Folate biosynthesis
Cytochrome P450
Butirosin and neomycin biosynthesis
Starch and sucrose metabolism
Cytoskeleton proteins
0.0000
0.0025
0.0050
0.0075
0.0100
Mean proportions
−0.0004
0.0000
0.0004
Difference in mean proportions
4.112554e−02
2.597403e−02
1.515152e−02
1.515152e−02
1.515152e−02
4.329004e−03
4.329004e−03
p−value(wilcox test)
